# Developing Disability-Focused Pre-Health and Health Professions Curricula

**DOI:** 10.1007/s10912-023-09828-8

**Published:** 2023-12-15

**Authors:** Rachel Conrad Bracken, Kenneth A. Richman, Rebecca Garden, Rebecca Fischbein, Raman Bhambra, Neli Ragina, Shay Dawson, Ariel Cascio

**Affiliations:** 1 College of Medicine, Northeast Ohio Medical University, Rootstown, OH USA; 2 Center for Health Humanities, Massachusetts College of Pharmacy and Health Sciences, Boston, MA USA; 3 SUNY Upstate Medical University, Syracuse, NY USA; 4 College of Medicine, Central Michigan University, Mount Pleasant, MI USA; 5 College of Education and Human Services, Central Michigan University, Mount Pleasant, MI USA; 6 Center for Bioethics and Social Justice, College of Human Medicine, Michigan State University, East Lansing, MI USA

**Keywords:** Ableism, Disability competency, Disability humility, Health professions education, Health justice, Simulated/standardized patients

## Abstract

People with disabilities (PWD) comprise a significant part of the population yet experience some of the most profound health disparities. Among the greatest barriers to quality care are inadequate health professions education related to caring for PWD. Drawing upon the expertise of health professions educators in medicine, public health, nursing, social work, and physician assistant programs, this forum showcases innovative methods for teaching core disability skills and concepts grounded in disability studies and the health humanities. Each of the essays offers practical guidance for developing curricular interventions appropriate for students at various levels of training and familiarity with disability to be implemented in classroom discussions, case-based learning, lectures, panels, and clinical simulations across the full spectrum of pre-health and health professions education.

## Introduction

### Rachel Conrad Bracken

The essays collected in this forum are motivated by overlapping, urgent concerns impacting healthcare practice and health professions education (HPE): unequal access and poor quality of healthcare for people with disabilities (PWD)^1^ and inadequate, inconsistent curricular approaches to teaching about disability in HPE. Neither of these concerns nor the proposed solutions are new. Health professions educators have warned that their students are unprepared to meet the healthcare needs of disabled patients since the early 2000s, and research continues to demonstrate the need for HPE focused on improving access to and quality of care for people with disabilities—what falls within the realm of knowledge and skills development—and dismantling ableism within healthcare settings (Shakespeare, Iezzoni, and Grace 2009; Campbell 2009; Cuff et al. 2016; Havercamp et al. 2021). Likewise, a robust body of literature documents persistent health inequities experienced by PWD, including barriers to accessing care; inferior quality of care, including less information about prevention and fewer screening tests; the lack of proper equipment to accommodate PWD in clinical spaces; increased risk of physical and mental health conditions, such as asthma, diabetes, and depression; and disparately poor outcomes, including premature death (Wen 2014; Mitra et al. 2022; World Health Organization 2023). In addition to physical, structural, communication, and knowledge barriers to accessing healthcare, PWD frequently encounter attitudinal barriers such as bias, stigma, and ableism, which, according to research published in a special issue of *Health Affairs* on the topic of “Disability & Health” just last year, “play a significant role in perpetuating health disparities for people with disabilities” (Lagu et al. 2022, 1394).

The link between HPE and health inequities is perhaps obvious; pre-health and health professions curricula must deliberately confront ableism—both within society at large and within the medical institutions, specifically—and provide students with opportunities to develop the knowledge, skills, and attitudes necessary to provide the highest quality of care to PWD. Nevertheless, most pre-health and health professions curricula continue to lack any, let alone standardized, disability-focused education (Ankam et al. 2019; Iezzoni et al. 2021), and there is little research establishing pedagogical and/or curricular best practices for disability education in the health professions (Ioerger et al. 2019). While there is a demonstrated need for disability education for pre-health and health professions students, many educators may have received minimal training or exposure to scholarly work in disability studies and either biomedical knowledge or clinical skills training targeted to caring for PWD.

Drawing upon the expertise of health professions educators in medicine, public health, nursing, social work, and physician assistant programs, this forum showcases innovative methods for teaching core disability skills and concepts grounded in disability studies and the health humanities. This conversation began with a panel discussion—“Transforming Disability-Focused Health Professions Education: Mobilizing Insight from the Intersection of Disability Studies and the Health Humanities”—presented at the Health Humanities Consortium’s annual conference in March 2023, and we are grateful for this opportunity to open up the conversation to a wider audience of health humanists and health professions educators.

Each of the essays offers practical guidance for developing curricular interventions appropriate for students at various levels of training and familiarity with disability to be implemented in classroom discussions, case-based learning, lectures, panels, and clinical simulations. First, Kenneth A. Richman presents techniques for introducing alternative ways of knowing and defining disability outside of a medical model steeped in considerations of impairment and pathology. Rebecca Garden then discusses strategies for navigating interdisciplinary and interprofessional approaches to disability education that honor the nuance and complexity of disability theory as well as the empirical demands of clinical practice. Describing the process of designing disability-focused simulated patient encounters for first-year medical students, Rachel Conrad Bracken, Rebecca Fischbein, and Raman Bhambra offer practical guidance for developing patient simulations, while Neli Ragina, Shay Dawson, and Ariel Cascio elucidate their multi-faceted Healthcare Education Engaging Disability Studies (HEEDS) program, modeling a robust interprofessional curriculum to conclude the forum. While certainly not comprehensive, we hope the pedagogical approaches described herein offer inspiration and transferable tactics for the development and implementation of disability curricula across the full spectrum of pre-health and health professions education.

## Breaking the Ice: Helping Allied Health Students Think About Disability Outside the “Medical Model”

### Kenneth A. Richman

Disability is a very natural and very human condition, but most of us do not learn to think and talk about it in helpful ways. For instance, although many disabled people are very satisfied with their lives (Albrecht and Devlieger 1999), physicians frequently underestimate the quality of life of people with disabilities (PWD), and when discussing living wills, it is common to hear the attitude “I’d rather be dead than disabled” (Reynolds 2017; 2018). When I teach Healthcare Ethics, a required course at my university, I try to address such attitudes and how little we—in our work as teacher-scholars or (future) healthcare professionals and in our everyday lives––are prepared to discuss them. Most of my students will work as nurses, as physician assistants, or in other allied health professions. A few will become physicians. Nearly all will provide care to disabled people. Learning to think and talk about disability accurately, respectfully, and responsibly is very important, and for many, it is very new.

Here, I describe how I teach allied health students about disability. My approach is informed by my disciplinary grounding in philosophy and my experience engaging with the autistic adults I have met through my research. Overall, I try to approach disability as an expected part of everyone’s lives while maintaining the humility appropriate to an ally without first-hand experience of disability.

I provide the bones of my approach expressed as imperatives, but putting the meat on those bones requires instructors to cultivate experiences and personal connections for themselves. I have not assessed my approach for effectiveness and am not sure what I would attempt to measure if I tried to assess it, but I believe that my approach helps students appreciate some of the simplicities and complexities around disability.

### Put Disabled People at the Center

Centering PWD can help break down our sense that disability is special, different, or foreign. I share images of the authors whose works we read to show that they are real people, not so different from the students, who are grappling with ideas. When we read Anita Silvers’s 1996 article “(In) Equality, (Ab) Normality, and the Americans with Disabilities Act,” we see Silvers in her wheelchair. She is an authoritative contributor to the discussion, not just a case study.

When using images, it is especially important to avoid triggering pity (seeing a disabled person as unfortunate or as suffering due to being disabled) or inspiration (admiration based on the sense that a disabled person must be especially brave or virtuous for doing what would be ordinary for someone who is not disabled). To address these two common reactions, I show a popular TEDx Talk by disabled comedian Stella Young (Young 2014). Young makes fun of the dominant view that disabilities are tragedies to be overcome through heroism and portrays her life as no more special or challenging than anyone else’s.

### Use Theory

I assign the aforementioned article because of the way Silvers presents the history, sociology, and economics of disability and connects a specific federal law in the United States to the two primary competing ways of thinking about disability. Some instructors may choose more recent literature with a richer or more subtle conceptual approach (e.g., Albrecht, Seelman, and Bury 2001); I find that a simple dichotomy can be effective for those with modest background in humanities. Indeed, while the understanding of disability as both embodied and socio-politically constructed is widely understood by those working within disability studies, this concept may be entirely new for health professions students thoroughly steeped in a medical model of disability.

Many readers will be familiar with two dominant disability models:

1. The Medical Model: Disability is (or is the result of) impairment or dysfunction in individuals.
2. The Social Model: Disability is “a state of society which disadvantages persons” (Silvers 1996, 215).

The social model separates impairment and disability so that—unlike under the medical model—someone with substantial impairment would not be disabled if they are not at a disadvantage. Disadvantages can arise from social attitudes and from the built environment. Insofar as disability is not in the world to be discovered but results from social decisions and attitudes, we say that it is “socially constructed.” The social model thus involves not just a different attitude to disability but a different understanding of the word *disability*. Per the social model of disability, impairments are a feature of “normal” human variation and not inherently disabling; an impairment is only disabling if or when the built and/or social environment fails to be inclusive of the full spectrum of human ability (Morris 2001).

The difference between the medical and social models can be highlighted by asking what each says about how to mitigate or eliminate disability. In the medical model, disability is a feature of individuals and can only be addressed by treating an individual’s impairment. According to the social model, disability is “*a problem which occurs as a result of social decision and is therefore subject to social correction*” (Silvers 1996, 215; original italics).

One point of Silvers’s article is to explain that the Americans with Disabilities Act (ADA) codifies the profoundly radical social model. Students tend to be familiar with the ADA, especially through the services offered to students with neurological differences. A 12-min companion video to the Netflix documentary *Crip Camp (How the ADA Changed the Built World)* (Newnham and LeBrecht 2020) can raise students’ awareness of how different things are since the ADA was signed by George W. Bush. It can be hard for those born after 1990 to imagine the built environment without curb cuts, ramps, and other accommodations required by the ADA, but the requirement for these is relatively new.

### Model Application of Theory to Cases

Using the vocabulary of disability theory to discuss familiar examples and generalizing to new cases are higher-order cognitive tasks, and it can be helpful to model them in the classroom. Rather than understanding the theories as different ways of perceiving the same situation or the same person, health science students can mistakenly think that some situations or people are examples of the social model while others are examples of the medical model. Once they are familiar with Stella Young and Anita Silvers, we can talk about how the different models give very different accounts of the same people and their circumstances. For instance, we note that per the medical model, Stella Young was disabled by osteogenesis imperfecta; in contrast, per the social model, she was disabled by things like buildings with stairs but no ramps or elevators. Providers who can acknowledge this perspective are better able to understand why many Deaf people reject cochlear implants and why so many autistic people do not feel the need to be “cured.” Providers with this insight can also help patients face a future with a progressive, disabling illness such as multiple sclerosis with less dread (Reynolds 2018).

### Make It Personal

Explaining disability in the context of people and places in your own life can make disability seem as ordinary as it is. After all, most people are disabled for at least part of their lives. Here, my experience spending time with autistic people for my research provides an advantage. I also tell a story about that time during the American Society for Bioethics and Humanities conference when I was lucky to join Anita Silvers and another colleague in search of dinner, and physical barriers kept us out of our first choice of restaurant. (We were ready to spend money in that restaurant, but we could not get the wheelchair in the door. How frustrating!) I can talk about the neurodivergent friends I met through my research on autism. Here is where each must find their own way based on their own lived experiences.

### Include Disability That Is Not Physical

No course will be able to cover all types of disability. Each person with a disability and disability community has its own vocabulary and concerns, but it is important, where possible, to include a discussion of cognitive disabilities as well as physical disabilities.

For those with appropriate knowledge (it takes exposure, reading, and time to develop a facility with the topic), autism can provide a useful example of cognitive differences that can result in disability. Attitudes expressed by autistic people, particularly those in the neurodiversity movement, can be especially helpful in disrupting assumptions. For example, people typically assume that autism is harmful to autistic people. “Neuroqueer” author Nick Walker describes how the neurodiversity paradigm challenges this. Walker describes the neurodiversity paradigm as promoting, among other claims, that:

The idea that there is one “normal” or “healthy” type of brain or mind, or one “right” style of neurocognitive functioning, is a culturally constructed fiction, no more valid (and no more conducive to a healthy society or to the overall well-being of humanity) than the idea that there is one “normal” or “right” ethnicity, gender, or culture. (Walker 2021, 33)

From a diversity perspective, disability is not a variance from “normal” but just another way to be normal. Disabled people cannot be different from a typical “us” unless the typical “we” are also different from disabled “them.” This is related to “the double empathy problem” (Milton 2012). We hear a lot about how autistic people have difficulty understanding non-autistic people. It is just as problematic that non-autistic people have difficulty understanding autistic people, but only one side tends to be seen as a disability.

### Question the Value of “Normal”

Learning to think, talk, and write about disability can require dislodging some habitual uses of the word *normal*. Students (and others) considering reasons for terminating a pregnancy or for euthanasia will sometimes cite the inability to have “a normal life.” When I am feeling cheeky, I might respond by asking whether the class thinks it is normal to wear bowties and go around talking about philosophy (referring, of course, to myself). The point is that normal is not the same as good or worthwhile. Not everyone wants to fit in or be typical.

Indeed, encouraging people to mask their disability or pass as typical (“normal”) can be oppressive. Walking without an assistive device, hearing through cochlear implants, or exhibiting neurotypical eye contact during social conversations can be uncomfortable and exhausting for those with relevant differences. If disabled people want to do those things, then they deserve support, but if PWD do not welcome them, then the pressure to conform can make lives worse rather than better.

### Question the Urge to “Cure”

Understanding disability can explode our ideas about what constitutes benefit and what is “medically necessary.” For instance, rather than wanting to be “cured,” many (not all) autistic people resonate with the statement: “There’s nothing wrong with me, I’m just autistic” (National Autistic Society 2023). Similar sentiments are shared by many culturally Deaf people and members of other disability communities. So, while impairments and atypical abilities may be in play, disability (in whatever sense) is probably not a patient’s chief complaint.

Learning to think about disability can relieve us of the assumption that what is good for a disabled patient is to mitigate the disability. We can learn to appreciate that providers (and anyone who cares for or about a person) may need to think and listen carefully before they can answer the question, *What’s the most appropriate care for this patient at this time?*

### Conclusion

As is characteristic of topics addressed in health humanities, disability can be explored at various levels of depth and detail and from multiple perspectives. In teaching my allied health students about disability, I try to show that disability is ordinary. I integrate images and the voices of disabled people, model the application of theory to cases, and tell about the disabled people I have known and their lives that are meaningful without being tragic or heroic. In the end, I want my students to think critically about how they use the word “normal” and question the idea that the only thing disabled patients need are interventions to make their impairments go away. I hope this provides some guidance to those seeking to integrate disability studies into their health professions teaching.

## Strategies for Advancing Disability Studies Perspectives in Healthcare Education

### Rebecca Garden

#### Navigating Differences When Building Collectives and Curricula

In the spring of 2023, a small ad hoc faculty task force organized to collectively address what most of us work on individually in different ways: the lack of focus on disability and ableism in the medical school curriculum. Some professors were new to the institution and/or to disability as a focus of pedagogy. Some of us have spent decades writing and teaching about—and advocating for—access, inclusion, and disability education. We were just beginning to build a consensus on curricular essentials, but the group was already breaking into camps. Some of us wanted to begin with the social model of disability as the foundational framework for understanding and addressing disability. A physician disagreed, arguing that, at least at the outset, the focus should be on clinical care rather than social issues. For her, the social model of disability belongs in a more advanced level of disability education. My colleague’s calculus of what constitutes essential disability education for healthcare providers is understandable. As a clinician who witnesses the physical barriers that her patients face every day inside the clinic and whose scholarly work underpins her expertise in disability in clinical care, she regards an analysis of social and structural barriers as secondary to the urgent need for equal access in clinical spaces to a disability-specific standard of care. Given disciplinary differences, how do health professions educators, who bring their scholarly and clinical expertise to bear on the question of what essential knowledge for practice is, navigate these roadblocks to develop a disability-focused curriculum that best prepares learners for clinical practice? What are effective strategies for educating clinical students and faculty about disability studies theories and perspectives?

Working collaboratively across these disciplinary barriers requires navigating the challenges of distinct training and sometimes radically different epistemologies. My strategy is to build on familiar concepts from health justice, such as cultural humility and structural competence, using that knowledge as scaffolding for more nuanced concepts such as disability cultural competency and the social and structural dimensions of disability. This strategy works in tandem with the health humanities imperative to scale out from a narrow focus on the individual and interpersonal to more fully analyze social and structural impacts on health and healing. Through a discussion of my pedagogical strategies, this essay provides a framework for bridging differences and building complex and nuanced understandings of disability among interdisciplinary faculty as well as students.

#### Building on Health Justice: The Political/Relational Model of Disability

Not wanting the conversation—or the task force’s momentum—to bog down, I began to explain how I teach my students to understand disability in terms of social and structural barriers, socio-political identity, *and* embodied difference, citing Alison Kafer’s definition of a political/relational model of disability (Kafer 2013). Kafer’s articulation of disability as socio-political as well as biological is fundamental to my pedagogy. For those unfamiliar with different models and theories of disability, however, this is not a quick conversation. Most of us are limited by “our shared disability illiteracy,” the fact that “most people don’t know how to talk about disability or how to be disabled” even though “disability is fundamental to being human” (Garland-Thomson 2017, 332; italics omitted). Further, Kafer’s paradigm dispenses with the reductive and thus easy-to-grasp medical model/social model binary, synthesizing critical disability studies theory to produce a working model that is subtle and complex. A strategy for navigating that complexity involves demonstrating how the political/relational model of disability maps onto health justice, a movement that centers community and foregrounds the impact of social and structural determinants of health, including the ways in which bias and discrimination cause access barriers, mistreatment, and neglect.

Like theorists working on other systemic inequities, Kafer critiques how the medical model “frames atypical bodies and minds as deviant, pathological, and defective, best understood and addressed in medical terms” (Kafer 2013, 5). Her political/relational model further identifies disabling structural barriers such as a lack of insurance coverage for assistive devices, sign language interpreters, or personal assistants, as well as social barriers such as stigma, bias, and the medical model itself. The World Health Organization’s definition of disability—a primarily medical model—similarly recognizes disability as the interaction among health conditions and social, structural, and environmental factors (World Health Organization 2001). Further, Kafer’s model emphasizes the significance of perceptions, attitudes, and structural supports for access needs and invites recognition that viewing “illness and disability [as] part of what makes us human” can productively co-exist with public health and healthcare efforts to avoid or diminish impairment or illness (Kafer 2013, 4). Agency and interdependence—key aspects of the political/relational model—locate authority over medical and public health interventions, as well as social and structural supports, in Deaf and disabled people. Mapping political and relational understandings of disability onto health justice is not only theoretical; many students, faculty, and staff, as well as patients, experience disabling access barriers, typically in contexts where diversity, equity, inclusion, and justice initiatives do not yet consistently include disability, if at all.

#### Scaffolding: Building Disability Studies onto Health Justice

My research and pedagogy offer a guide to analyzing social and political dimensions of disability through narrative perspectives, for example, exploring diagnosis and medicalization through narratives such as Eli Clare’s *Exile and Pride* (2009) and *Brilliant Imperfection* (2017). To facilitate students’ understanding of disability as the embodiment of structural inequities, I build on the social focus in narratives—such as the impact of toxic norms and master narratives on identity—with structural analyses. Learners explore inequities and disparities at the structural level through stories: viewing TikTok videos about policies that restrict access to assistive devices and interviewing disabled people and healthcare providers about navigating those policies through DIY (do-it-yourself) design hacks and policy change. Through the scaffolding process, students recognize these narratives—as well as scholarly and activist writing on disability justice, disability design, and collective access—as disability-specific strategies for structural competence (Mingus 2010; Hamraie 2013; Berne et al. 2018).

These political and relational approaches to disability build on familiar concepts: structural violence, cultural humility, and structural competence. I have used this approach whether designing an entire ethics course for nursing and health professions or creating a single unit on disability for the first-year medical students’ bioethics course. For the standalone session in the first-year students’ case-based bioethics course, I scaffolded health humanities and disability studies analytical tools and strategies onto required ethics and health justice topics and concepts, enabling learners’ mastery of the more nuanced concepts. Assigning the same prerecorded introduction to disability and a unique cluster of short readings and media to each student enables them to educate each other about concepts, tools, and policies such as the social model of disability, disability cultural competence, universal design, and the Americans with Disabilities Act. I wrote the case narrative to encourage students to engage with structural and social complexities. The case involves a wheelchair user navigating structural ableism—transportation barriers and barriers in the built environment that contribute to substandard clinical care—as well as social forces such as bias and inadequate knowledge and skills. The patient’s frustration and anger might lead some students—and faculty—to frame the issue as one involving a “difficult patient.” The teaching guide cues the students to reflect on their responses to emotion and explore the impact of bias and structural inequities, encouraging them to recognize how “choices are structured by oppression” (McBryde Johnson 2003). The teaching guide invites students to discuss strategies for access and inclusion and topics such as the limits of empathy and whether and how providers should disclose their chronic illness and disability status. In my own classroom, I remind students that I identify as disabled and that others likely do as well.

#### Weaving Disability Studies into the Curriculum and Culture

Education that addresses ableism and centers disability culture and justice must escape the confines of a special topics lecture or elective. Integrating disability education can be strategic, seeding disability culture at the institution. One strategy I practice to engage learners who may not have a pre-existing interest in the required and elective disability curricula is to embed disability in a series of co-curricular university-wide panels that I moderate on a range of health justice topics. The Disability and Healthcare panel includes disabled designers, educators, students, and community organizers. A presenter for the Refugee Health panel is a Deaf New American/refugee interpreter and community organizer. Whenever I can, I include Deaf and disabled colleagues on panels that are not explicitly about disability and Deaf culture, allowing recognition of disability and Deafness as intersectional identity and experience, a rebalancing that enables students to witness the ways that disability and Deaf epistemologies map onto other forms of experience, knowledge, and expertise and offer invaluable perspectives on public health and healthcare.

In addition to scaffolding disability and Deaf health onto health justice topics, I recruit students who may, at first, be more intrigued by interdisciplinary learning than disability. I designed a two-week co-curricular session that teamed up healthcare and landscape architecture students to document accessibility on the health science campus. Led by disabled and elder community activists, the teams delved into disability as a social and structural effect by documenting access barriers in classrooms, campus life, and public spaces on our campus. The disability focus is built on nondisabled students’ experiences of barriers to access and inclusion in the academic environment.

The elective I teach for public health, medical, and nursing students attracts students interested in narrative- and arts-based approaches to health disparities; only a few students enroll for the focus on disability studies approaches. Here, too, scaffolding key concepts such as disability justice and mutual aid, the political/relational model of disability, and disability narrative identity onto familiar ones such as structural violence and cultural humility helps students to locate disability within a broader understanding of health justice (Scully 2008).

Students analyze health disparities through interviews, PhotoVoice, TikTok, Twitter, street art, zines, and graphic medicine. Their presentations and reports model disability accessibility through multimodal formats such as image/text zines linked with audio files. We take field trips to recognize the importance of place in relation to health and identity and to get to know Deaf and disabled people where they live, work, and socialize. The course concepts help students attune to the embodied impacts of structural inequities, scope into those experiences at the granular level, and scale out to identify the structural influences. One semester, we focused on the impact of pandemic-related food access barriers on Deaf and disabled people; another semester, we studied the pandemic-related policies and practices that disproportionately burden disabled, chronically ill, and other marginalized communities. This year, my course explores the intersectionality of HIV/AIDS, disability, austerity, and structural racism.

My scaffolding strategy for teaching works in conversations with colleagues, as well. I can explain the more nuanced political/relational model by identifying similar elements in the World Health Organization’s definition of disability or the biopsychosocial model. The scaffolding is also part of the interdisciplinary work of the health humanities. At every scale of teaching about disability, I weave disability studies concepts together with social science theory and health humanities to scale out from the individual sphere of experience and identity to the social and structural spheres through an anti-ableist health humanities pedagogy. The interdisciplinary nature of the health humanities prepares us to bridge differences in education and training—clinical medicine versus humanities and social science—that may pose challenges to coordinating curriculum development. As advocates united in our commitment to improving disability education at our institution, we must adapt and advance, even when we initially stall out due to clashing perceptions about what, in essence, constitutes disability. Strategic and patient communication and cross-disciplinary collaboration will shape disability education in our curricula and help to integrate disability into the culture of our institutions.

## Integrating Disability-Focused Simulated Patient Encounters in Health Professions Education: Practical Guidelines for Design and Implementation

### Rachel Conrad Bracken, Rebecca Fischbein, and Raman Bhambra

In January 2022, we (the authors) began the process of designing, piloting, and finally implementing new, disability-focused simulated patient training for first-year medical students at Northeast Ohio Medical University (NEOMED). Standardized and simulated patient encounters are one way to increase student knowledge and awareness, reduce stigma, and improve attitudes toward individuals with disabilities (Long-Bellil et al. 2011; VanPuymbrouck et al. 2017). Research further suggests that providing these experiences for undergraduate medical students can help hone crucial listening and communication skills with a patient population that has historically faced discrimination and unequal access within the United States healthcare system (Iezzoni et al. 2021). While some United States medical schools have successfully integrated patient simulations into their disability curricula, this practice is not standard (Eddey, Robey, and McConnell et al. 1998; Long-Bellil et al. 2011). Moreover, little information exists regarding best practices and resources for medical schools regarding how to partner with disability communities to conduct outreach and recruitment; ensure accessibility of space and materials; develop and/or reframe simulated cases; and educate patient actors, students, simulation staff, and faculty. To help address these gaps, this essay outlines strategies for determining learning objectives for disability-focused simulations, developing case narratives, recruiting and training simulated patients with disabilities, and evaluating the effectiveness of simulated patient encounters to develop cultural humility and communication skills.

#### Simulated Versus Standardized Patient Encounters

Simulated and/or standardized patient encounters are a well-established component of health professions education (HPE). Broadly conceived, simulated patient encounters allow students to practice clinical skills with trained actors and to evaluate these skills. This includes semi-structured interviews and partially scripted simulations, in which patient actors may or may not utilize elements of their own personal medical and social history, and standardized encounters, in which patient actors provide consistent verbal and behavioral responses according to a detailed, comprehensive, and meticulously calibrated script (Adamo 2003). Thus, standardized encounters are uniform and often evaluative, used to *test* clinical skills, whereas simulated encounters may also be formative, designed to *teach* clinical skills. In this essay, we use the terms *simulated patient* (SP) and *simulated patient encounters* (SP encounter) to refer to non-standardized, loosely scripted encounters as opposed to uniform, strictly standardized experiences.

When utilized as a component of disability-focused HPE, we strongly recommend the use of disabled SPs and discourage uniformly standardized cases. The use of nondisabled SPs to portray people with disabilities (PWD) invites reductive, stereotypical, and otherwise problematic depictions of disability (Havercamp et al. 2021). “Furthermore,” Havercamp and colleagues (2021, 7) attest, “actors without disabilities are not credible in portraying aspects of disability such as atrophied muscles, poor head control, deafness, blindness, contractures, spasticity, dysarthric speech, or the use of communication devices or interpreters.” Similarly, a standardized encounter flattens disability, falsely suggesting, for instance, that all blind folks or wheelchair users experience visual or mobility impairments in the same way. A partially scripted simulation allows SPs to authentically portray their disability and honors the rich diversity within disability communities. It that exposes students to a wider range of presentations and perspectives while providing the opportunity to practice clinical skills, proper equipment use, and assessment execution (VanPuymbrouck 2017). Moreover, previously published research demonstrates that early and frequent encounters with PWD can both combat ableist attitudes and aid students’ acquisition of clinically relevant knowledge and skills (Santoro et al. 2017), further prioritizing interactions with PWD as SPs.

#### Determining Learning Objectives for Simulated Patient Encounters

The process of determining learning goals for SP encounters is collaborative, engaging three essential stakeholder groups: faculty, who must ensure that simulations align with course objectives and longitudinal educational program objectives; students, who crave practical, clinically relevant material; and PWD, for whom the stakes of these curricular innovations are the highest. To address the needs of all stakeholders, we consulted a growing body of published literature on “disability competencies” in HPE, conducted focus groups with members of local disability community organizations and recent NEOMED College of Medicine and College of Pharmacy graduates, and compensated expert consultants from the disability communities represented in our simulations to refine learning objectives appropriate for first-year medical students. Because we do not identify as disabled, despite close personal connections to the disability community, it was especially important for us to heed disability activists’ rallying call “Nothing about us without us” via focus groups and partnerships with expert consultants from our local disability communities to identify critical gaps in the provision of healthcare for PWD.

In the latter half of 2022, we facilitated interviews and focus groups with 32 members of local disability community organizations (participants identified as blind or low vision, mobility impaired, or having an intellectual disability) and 10 NEOMED alumni to identify healthcare providers’ perceived knowledge and skills gaps relative to providing care to PWD. We recruited focus group participants from disability community organizations in our region, and snowball sampling further expanded our participant pool. Interview and focus group transcripts were inductively coded using thematic analysis to identify central themes and shared concerns among participants, then distilled into a comprehensive list of learning goals that reflected what students wished they had learned and what PWD wished their providers knew about providing healthcare to PWD. These institutionally specific learning goals were then aligned with the list of Core Competencies on Disability for Health Professions Education established by the Alliance for Disability in Health Care Education (ADHCE)—an essential resource for anyone looking to develop disability-focused curricula across the full spectrum of HPE (ADHCE 2019; Havercamp et al. 2021). Our learning objectives were further refined in conversation with expert consultants, as well as faculty overseeing the course in which our SP encounters are housed and simulation center staff. Because these encounters were designed for first-year medical students, our focus was on formative learning rather than summative evaluation of students’ clinical and interpersonal skills; curricular and community stakeholders collectively agreed upon learning goals and session objectives provided in Table 1.

**Table 1:** Learning Goals and Session Objectives

Learning Goals	Upon completion of this exercise, students will:	1. Gain familiarity and become comfortable treating patients with disabilities as they would non-disabled patients
		2. Demonstrate mastery of general principles of professionalism, communication, and respect for patients
		3. Recognize optimal health and quality of life from the patient’s perspective
Session Objectives	By working with simulated patients (SPs) with disabilities, students will:	1. Practice interacting with and providing care to patients with disabilities
		2. Increase their comfort and confidence working with patients with disabilities

#### Developing Case Narratives

To ensure that our disability-focused SP encounter, which replaced an existing simulation in the first-year medical curriculum, fulfilled both the original session objectives and disability-specific learning goals, we chose to modify a previously used case in collaboration with course faculty, simulation center staff, paid consultants with lived experience of disability, and second-year medical students. The case was generic—the patient presented with a headache—and the symptoms and presentation were standardized. Since our SPs represented multiple disabilities—the SPs who participated in this encounter were blind or low vision, Deaf/hearing impaired, or had Down syndrome—the case could not be fully standardized. To honor the diversity of perspectives and experiences within the disability community and specific subpopulations, our case was written such that SPs provided their own medical history and psychosocial background, with a few clinically significant exceptions (e.g., SPs were to report no allergies and no history of migraines) to maintain the fidelity of the symptom presentation. To streamline the encounters and ensure students experienced similar simulations, we asked SPs to limit their responses to past medical history and family medical histories to the two most recent or most significant events and permitted them to omit or fabricate elements of their history to respect their privacy.

Consistent with best practices (Long-Bellil et al. 2011; Billon et al., 2016; Sarmiento et al. 2016), students, faculty, simulation staff, and expert consultants from the disability communities represented were invited to provide feedback and suggest revisions as the case was iteratively refined and piloted. Our team assessed the case for timing, flow, clarity, clinical accuracy, and accessibility of both our simulation center and all preparatory materials for SPs. Ultimately, case design must be guided by the learning goals established for a specific simulation to best meet the needs of students and the communities represented. Including PWD in the development of simulated patient encounters via focus groups to determine learning objectives and paid consultants to guide case development prioritizes the healthcare needs of the population in question by decentering a medical model of disability and honoring the expertise of PWD. Moreover, it aligns with a patient-as-educator approach (Karazivan et al. 2015) and models an inclusive, participatory approach to curriculum development that elevates the experience and expertise of PWD.

#### Recruiting and Training Simulated Patients

Though some simulations may be focused on a particular type of disability, such as mobility impairment or autism, we sought a pan-disability pool of SPs. Partnering with local disability community organizations proved to be the most effective way to recruit SPs, as our liaisons in these organizations lent trust and credibility. We specifically identified community organizations with a focus on the performing arts, including a mixed-ability dance troupe, a Deaf theater company, and an improvisational acting group for folks with developmental disabilities, which allowed us to reach individuals interested in acting and familiar with memorizing and performing from a script.

Our simulation center staff led SP training but worked closely with faculty and expert consultants to ensure the accessibility of training materials and the physical space of the simulation center. Together, we performed an “accessibility audit,” which allowed us to identify potential barriers to inclusion and subsequently work to provide accommodations, such as sighted guides for blind participants and American Sign Language interpreters for Deaf/hearing-impaired participants. We strongly encourage curricular developers to consult individuals with lived experience of disability to ensure the unique access needs of site-specific SP populations are met.

#### Preparing Students and Providing Feedback

If they are to successfully ameliorate the ableist discrimination frequently encountered in biomedical spaces, SP encounters must be grounded and contextualized through theoretical training in disability studies and the health humanities (Campbell 2009). Put simply, students must be prepared to make the most of SP encounters and given space to consciously debrief the experience. To prepare students to engage with disabled SPs, we presented an introductory lecture on disability theory followed by a patient panel featuring representatives from each of the disability communities to be included in patient simulations; panelists shared their experiences, good and bad, seeking healthcare and provided students with strategies for engaging PWD with respect and compassion. A tip sheet summarizing advice offered by panelists and an example SP video were distributed to students. An additional didactic session aimed at alleviating student anxiety emphasized the formative nature of the exercise and offered insight from three second-year medical students who assisted in the case design and pilot.

After each encounter, SPs rated students on elements of communication, etiquette, and attitude aligned with the learning goals and session objectives. Students also received feedback from peers and faculty during small group seminars in which they reviewed recordings of their own and their groupmates’ SP encounters. Students were intentionally assigned to SPs such that at least one member of each seminar group interacted with each of the disability communities represented, allowing all students to observe and discuss the unique needs of each of these populations. (For example, a student who had been assigned to a blind SP would have the opportunity to review the encounter between a peer and a Deaf SP.)

#### Evaluating Impact

This has been a powerful experience for all those involved, including SPs, students, and faculty. Formal evaluation activities demonstrating this impact include pre- and post-simulation surveys completed by students, which document decreases in stigma, increases in confidence, and increases in disability-related knowledge. Qualitative feedback from students reveals similar themes, including the positive impact of exposure, better understanding of the barriers faced by PWD when attempting to access healthcare, increased confidence to treat those with disabilities, the realism of the encounter compared to other simulated experiences, and how similar PWD are to non-disabled patients.

We have also surveyed SPs regarding their experiences and their responses were primarily positive, demonstrating satisfaction with the accessibility and support provided by the simulation center and interest in serving as SPs in the future. We received suggestions for improvement, such as additional reminders for students to use simpler terms when interviewing SPs and logistical suggestions regarding the timing and length of simulation days. As we work to replicate and expand this activity in future years, we will continue to evaluate the impact this has on our university and local disability communities. Our five-year, longitudinal assessment plan is designed to determine whether SP experiences can lead to long-term changes in disability-related attitudes and perceptions, data we hope will encourage the integration of disability-focused SP encounters in more HPE programs.

### Conclusion

Intended to be descriptive rather than proscriptive, we hope that the experiences and suggestions shared here can be adapted and implemented in other HPE programs to suit the needs of various institutions and student populations. Adding simulated patient encounters to our medical school’s existing disability-focused curricula—which includes lectures on ethics, law, culture, and disability theory; workshops; patient and provider panels; and narratively-based small group discussions—offers students an opportunity to gain familiarity with PWD and to begin to explore the nuances of providing competent and compassionate care. Moreover, applying what is learned in didactic sessions to simulated clinical settings illuminates the clinical utility of disability theory and health humanities approaches to disability education, encouraging students to challenge a wholly medical model of disability and their own entrenched biases or misperceptions. Indeed, research suggests that “early and frequent encounters with people with disabilities aid student’s knowledge, skills, and attitudes about providing care for such patients” (Santoro et al. 2017, 757).

Most importantly, perhaps, SP experiences can help to cultivate disability humility—the recognition that one’s understanding of disability culture and disabled experience will only ever be partial (Reynolds 2018)—and respect for the expertise of PWD in medical spaces. Beyond training future providers, including PWD in the design, refinement, and evaluation of SP cases invites members of the disability community to share authority with healthcare providers and medical educators to collaboratively establish learning goals and evaluate medical performance, thereby subverting conventional power imbalances and reshaping the contours of disability in medical spaces.

## An Interprofessional Guide to Improving Healthcare for People with Disabilities: The Healthcare Education Engaging Disability Studies (HEEDS) Program

### Neli Ragina, Shay Dawson, and Ariel Cascio

To address the well-documented need to provide disability-specific training within healthcare education (McColl et al. 2008; Werner et al. 2017), we have developed Healthcare Education Engaging Disability Studies (HEEDS), an interprofessional educational program grounded in the biopsychosocial theoretical foundation of the International Classification of Functioning (ICF) as defined by the World Health Organization. The ICF combines the best of the medical model of disability—clear treatment paths for functional impairment—with the social model of disability to address barriers within an individual’s environmental and personal domains with a goal of full participation in society (World Health Organization 2001). HEEDS aims to address healthcare disparities and provides important opportunities for health professions students to gain early exposure to key knowledge and skills in caring for people with disabilities (PWD) and to build meaningful connections with local disability communities (Table 2).

**Table 2:** HEEDS Program outline

Activity	Disability Studies Seminar		Essential Clinical Skills (ECS) mock patient encounter	Initial Clinical Experience (ICE) with disability community partners	Objective Structured Clinical Examination (OSCE)	
Learners	1st and 2nd Year Medical Students	Health Professions Students	1st and 2nd Year Medical Students	1st and 2nd Year Medical Students	3rd Year Medical Students	Health Professions Students

HEEDS began with a needs assessment (Hamilton et al. 2022). Interviews and focus groups found that people with physical and developmental disabilities in our local communities experience (1) a lack of patient-centeredness that impedes the quality of care, (2) inadequate communication that marginalizes patients within the clinical encounter, and (3) accessibility barriers that interfere with navigating the healthcare system. These findings reflect and nuance the results of previous studies (McColl et al. 2008; Werner et al. 2017), which emphasize the need for an effective educational intervention that can serve as a guide for healthcare professionals in academic and non-academic settings who strive to improve healthcare delivery for PWD. This essay offers a description of the development and preliminary outcomes of the HEEDS program to inform health profession educators interested in implementing an evidence-based curriculum focused on disability and medicine.

### HEEDS Program Advisory Board

The HEEDS program is guided by a community advisory board whose members all have personal or professional experiences with disability. This board has consisted of (1) external academic advisors Lisa Iezzoni, MD (Harvard) and Raymond Curry, MD, FACP (University of Illinois Chicago College of Medicine); (2) representatives from local disability organizations, including Peckham, Inc. (a vocational rehabilitation non-profit), Special Olympics, Michigan Career and Technical Institute, Mid-Michigan Industries, Michigan Department of Health and Human Services, Community Mental Health of Mid-Michigan, and Riley Children’s Foundation in Indiana; and (3) individuals with personal lived experience of disability. Many board members have both professional roles and lived experience of disability.

The HEEDS Advisory Board provides input on the study and the program design. The program involves volunteer projects, a seminar series, and standardized patient encounters. The Advisory Board recommends and reviews research instruments, volunteer partner agencies, current issues in healthcare that should be a part of students’ learning, and typical problems that PWD encounter when interacting with medical professionals. The Advisory Board also works with the College of Medicine to recruit people with lived experience of disability to be standardized patients (SPs) and co-develops the scenarios for disability-specific SP encounters.

### Initial Clinical Experience (ICE) Volunteer Projects

The ICE projects allow students to interact directly with PWD and practice their clinical skills through hands-on experiences embedded within the medical school curriculum. Learning objectives include (1) improving awareness of disability-specific social and access issues, (2) increasing students’ knowledge of community organizations that support PWD, and (3) fostering compassion and communication skills through peer-to-peer activities. For example, medical students taught CPR (cardiopulmonary resuscitation) to Special Olympics athletes. Athletes learn skills to respond to emergencies, and students increase their familiarity with interacting with people with intellectual disabilities. The rationale for these experiences is not only to enhance students' education and understanding of health disparities that PWD face, but also to provide hands-on experiences that will prepare them to work with PWD.

### Disability and Medicine Seminar Series

The Disability and Medicine Seminar Series aims to expose students to disability studies and discuss both legal obligations and best practices in working with disabled patients. Five one-hour modules address (1) problem areas in disability and medicine, (2) implicit biases in disability and medicine, (3) models of disability, (4) patients with physical disability, and (5) patients with intellectual disability and Autism. Each module consists of a videotaped interview with a patient or medical professional who has lived experience with disability. These interviews leverage storytelling to introduce students to several community members impacted by disability, some of whom are also SPs who interact face-to-face with students later in the HEEDS program. Storytelling is especially important in the context of working with patients with disabilities, as it aids in combating stigma, stereotypes, misconceptions, and prejudices that often surround disabilities and highlights areas where improvements in healthcare are needed. Embedding the lived experiences of PWD in the curriculum assists students in perceiving medical diagnoses through the lens of the social and biopsychosocial models of disability. This perspective shift results in the healthcare provider making accommodations for PWD while improving their own interaction skills.

The seminar series introduces students to key topics that are important in working effectively with disabled patients in the healthcare setting. Although medical and other health professions students are trained in diagnostics and treatment, their curricula typically focus exclusively on the use of the medical model of disability (Evans 2004). HEEDS seminars offer alternative modes through which to comprehend disability via the social and biopsychosocial models of disability (Shakespeare 2010; World Health Organization 2023). Throughout the series, students also engage with discussions about person-first versus identity-first language; empowerment, engagement, and empathy as specific approaches with patients/clients; health disparities; and psychosocial aspects related to disability, stigma, and social marginalization. Students view a recorded interview with a disabled person during each of the five respective lectures. For example, during the lecture on intellectual disability and Autism, one interview consists of a physician assistant who has an adult child who is Autistic with high support needs. This medical professional, with lived experience as a parent of an Autistic child, has become a go-to service provider for Autistic patients and provides insights into providing exceptional care, including specific approaches within the clinic. A second lived experience interview consists of a married couple that has physical disabilities and utilizes power wheelchairs for their mobility needs. They tell a maternity-specific story of the birth of their son (without a disability) in the hospital setting. Shortly after birth, the medical team insisted that the child be placed in foster care despite the couple’s ability to seek outside support to help care for the child in their home. Medical professionals failed to mention any concerns in the nine months of medical care leading up to the birth when the couple could have prepared for any concerns raised. After much discussion, the couple was able to take their child home with support in place and have been successful in providing a nurturing and developmentally appropriate home life. The mother also shares how, leading up to the birth of her child, she was never physically examined or weighed, thus receiving drastically different care than is the routine standard of practice directly due to her disability. Such stories captivate students’ attention and provide a platform for rich discussion of what went wrong and how they could avoid similar situations in their future practice by providing better accommodations and understanding.

### People with Physical and Intellectual Disability as Standardized Patients

The HEEDS program includes two SP cases, one for a patient who uses a wheelchair and a second for a patient with an intellectual disability and their care partner. With the help of the Advisory Board, the College of Medicine has hired individuals with lived experiences of these disabilities to work as SPs in these (and sometimes other) cases. The first case consists of a 45-year-old patient named Mel who has a physical disability and uses a wheelchair. The patient presents with a hand wound from cutting vegetables several weeks prior and is concerned that it has not healed. The patient also complains of not having an accessible exercise facility in town and thus has not been able to maintain an active lifestyle. The second case includes a 20-year-old patient named Jo or Joe Smith, who has an intellectual disability and presents with an earache after swimming recently. The patient comes to the office with their adult care partner and complains of not being able to participate in community leisure activities, including swimming, due to the pain experienced in their ear. The patient would like to return to their active lifestyle as soon as possible. In both cases, the goal of the patient encounter is to hone personal interaction skills and improve comfort levels in working with patients with disabilities. Although the students are also concerned with the medical outcomes of the encounter (e.g., finding out why the wound has not healed and curing the earache), the interaction with PWD is most critical to the outcomes of the program.

Prior to medical students working with the SP actors, they are trained by an expert on the implementation of an accessible physician’s office room that includes a weight scale to accommodate wheelchair users, an examination table that moves up and down, and an accessible sink for healthcare professionals who use wheelchairs to wash their hands. After the medical students participate in the SP cases, a large group debrief is completed with the roughly 100 medical students from the first-year class, as well as the SPs and care partners. During this debrief, best practice approaches are described by the leaders and actors, as well as real-life experiences that the disabled actors have experienced personally in the past. Medical students have an opportunity to ask questions and share their experiences within the SP encounters and to receive feedback and guidance. This debrief is innovative and unique in that it is rare for the entire class cohort to hear directly and synchronously from PWD regarding their lived experiences with barriers within the healthcare system.

SP cases are integral to the HEEDS program not only to provide hands-on experience in working with PWD but also to train students to tailor treatment plans and communication to individual patients’ needs and preferences and increase their clinical competency in diagnosing, treating, and supporting PWD. Ultimately, these experiences will shape future healthcare professionals who can contribute to the creation of inclusive healthcare environments. This includes ensuring that healthcare facilities are accessible and welcoming to PWD.

### Conclusions and Outcomes

Disability studies has an important role to play in healthcare professions education. Exposure to disability studies frameworks provides a necessary balance to the medical model in health professions curricula. The HEEDS program incorporates disability studies not only in its content but also in its form. It derives from community needs and connects students with PWD and disability-focused community organizations both inside and outside the classroom. People with lived experience of disability guide the project through the Advisory Board. They serve as advisors, storytellers (in interviews shown during the seminar series), and standardized patients. Implementing experiences that provide direct exposure to PWD addresses the recommendation by Santoro et al. (2017). Recent research on medical student participation in the HEEDS program included a pre- and post-survey using a validated disability-specific attitude survey (Symons et al. 2012). Findings demonstrated a 15–20% improvement in healthcare professionals’ attitudes toward patients with disabilities in terms of feeling comfortable around and performing physical exams on patients with both physical and intellectual disabilities, as well as improved comfort interacting with disabled patients in everyday settings.

The HEEDS program focuses primarily on physical and intellectual disability, including through two SP scenarios. These foci derive from local community partnerships but certainly do not represent the whole spectrum of human diversity and disability. We recommend that future work should apply the basic approach of this model—including needs assessment, student community engagement, co-created SP scenarios, SPs with lived experience, and a debrief with those SPs and hiring actors representing other experiences such as auditory, visual, or psychological disabilities.

## Data Availability

The data that support the findings of this study are available from the corresponding author, NR, upon reasonable request.
