# Correction: Developing Disability-Focused Pre-Health and Health Professions Curricula

**DOI:** 10.1007/s10912-024-09917-2

**Published:** 2024-11-22

**Authors:** Rachel Conrad Bracken, Kenneth A. Richman, Rebecca Garden, Rebecca Fischbein, Raman Bhambra, Neli Ragina, Shay Dawson, Ariel Cascio

**Affiliations:** 1 College of Medicine, Northeast Ohio Medical University, Rootstown, OH USA; 2 Center for Health Humanities, Massachusetts College of Pharmacy and Health Sciences, Boston, MA USA; 3 SUNY Upstate Medical University, Syracuse, NY USA; 4 College of Medicine, Central Michigan University, Mount Pleasant, MI USA; 5 College of Education and Human Services, Central Michigan University, Mount Pleasant, MI USA; 6 Center for Bioethics and Social Justice, College of Human Medicine, Michigan State University, East Lansing, MI USA

**Correction: Journal of Medical Humanities (2023) 44:553–576**

10.1007/s10912-023-09828-8

In the recently published article, the references to “Alliance for Disability in Health Care Education” or ADHCE are mistakenly included as “Alliance for Disability in Health Professions Education” or ADHPE.

The original article has been corrected.

